# Potential of Polyethyleneimine as an Adjuvant To Prepare Long-Term and Potent Antifungal Nanovaccine

**DOI:** 10.3389/fimmu.2022.843684

**Published:** 2022-05-16

**Authors:** Zhao Jin, Yi-Ting Dong, Shuang Liu, Jie Liu, Xi-Ran Qiu, Yu Zhang, Hui Zong, Wei-Tong Hou, Shi-Yu Guo, Yu-Fang Sun, Si-Min Chen, Hai-Qing Dong, Yong-Yong Li, Mao-Mao An, Hui Shen

**Affiliations:** ^1^ Department of Pharmacology, Shanghai Tenth People’s Hospital, Tongji University School of Medicine, Shanghai, China; ^2^ Department of Clinical Laboratory Medicine, Shanghai Tenth People's Hospital, Tongji University School of Medicine, Shanghai, China

**Keywords:** polyethylenimine, nanoparticles, fungal infections, long-lived plasma cell, long-term protection

## Abstract

**Background:**

*Candida albicans* infections are particularly prevalent in immunocompromised patients. Even with appropriate treatment with current antifungal drugs, the mortality rate of invasive candidiasis remains high. Many positive results have been achieved in the current vaccine development. There are also issues such as the vaccine’s protective effect is not persistent. Considering the functionality and cost of the vaccine, it is important to develop safe and efficient new vaccines with long-term effects. In this paper, an antifungal nanovaccine with Polyethyleneimine (PEI) as adjuvant was constructed, which could elicit more effective and long-term immunity *via* stimulating B cells to differentiate into long-lived plasma cells.

**Materials and Methods:**

Hsp90-CTD is an important target for protective antibodies during disseminated candidiasis. Hsp90-CTD was used as the antigen, then introduced SDS to “charge” the protein and added PEI to form the nanovaccine. Dynamic light scattering and transmission electron microscope were conducted to identify the size distribution, zeta potential, and morphology of nanovaccine. The antibody titers in mice immunized with the nanovaccine were measured by ELISA. The activation and maturation of long-lived plasma cells in bone marrow by nanovaccine were also investigated *via* flow cytometry. Finally, the kidney of mice infected with *Candida albicans* was stained with H&E and PAS to evaluate the protective effect of antibody in serum produced by immunized mice.

**Results:**

Nanoparticles (NP) formed by Hsp90-CTD and PEI are small, uniform, and stable. NP had an average size of 116.2 nm with a PDI of 0.13. After immunizing mice with the nanovaccine, it was found that the nano-group produced antibodies faster and for a longer time. After 12 months of immunization, mice still had high and low levels of antibodies in their bodies. Results showed that the nanovaccine could promote the differentiation of B cells into long-lived plasma cells and maintain the long-term existence of antibodies *in vivo*. After immunization, the antibodies in mice could protect the mice infected by *C. albicans*.

**Conclusion:**

As an adjuvant, PEI can promote the differentiation of B cells into long-lived plasma cells to maintain long-term antibodies *in vivo*. This strategy can be adapted for the future design of vaccines.

## Introduction

*C. albicans* is one of the most common causative agents of fungal infections worldwide, especially in immunocompromised individuals. Even with proper antifungal drug treatment, the mortality rate of *C. albicans* infection remains high at 40–50% (1, 2). Increasing drug resistance by fungal pathogens and the decrease of new therapeutic agents are the major hurdles to cover. Thus, antifungal vaccines for specific populations are currently considered the most attractive strategy (3). Vaccines have played an important role in public health by controlling infectious diseases and extending life expectancy. Despite the importance of vaccinology, we are still in the exploratory stages of how we can achieve better protective efficacy and develop long-term immunity through improved vaccine design (4). The protective efficacy and durability of antibodies are two important indicators of evaluating vaccine efficacy. It is beneficial if a vaccine could elicit more effective and long-lived immunity with fewer vaccinations. First, longer-lived immunity could provide people with longer protection without fear of losing their antibodies. Second, production of cheap and effective vaccines is particularly important for cost savings, especially in developing countries (5).

Studies provide the possible underlying mechanistic insight into how long-term antibody responses are maintained; long-lived plasma cells (LLPCs) appear to sustain antigen-specific antibody levels (4). LLPCs are believed to differentiate from antigen-specific B cells in germinal centers (GC) reaction. In the absence of LLPCs help, some vaccines elicit only short-lived immunity and no immunological memory. At the same time, studies have shown that antifungal vaccines can induce immune response in mice, but for various reasons, further studies have not been carried out (6). One of the obstacles is the full domain of the antigenic protein, which tends to produce ineffective antibodies (7). It has been shown that patients recovering from invasive candidiasis have antibodies produced against specific fragments of Hsp90, rather than the antibodies of full length. Therefore, we believe that immunization with full-length Hsp90 activates unnecessary antibodies (8–10). Also, no evidence has been proposed to prove whether vaccines can induce long-term antibody in mice. The main barriers are the weakness of antigens and the absence of suitable adjuvants to stimulate the differentiation of B cells into LLPCs. Therefore, the introduction of highly effective adjuvants to maximize the efficacy of the vaccine is a promising strategy. With the development of nanotechnology, nanovaccines have attracted more attention. Owing to the unique characteristics, nanovaccines showed remarkable vaccine efficiency in stimulating or modulating the immune response *in vivo* (11, 12). Polyethylenimine (PEI) is one of the well-known cationic polymers. Increasing evidence has shown that PEI could act an important role as adjuvants in nanovaccines (13–15). In our study, PEI was developed as an adjuvant with a novel function: stimulating B-cell differentiation *in vivo*. Therefore, we developed an efficient and safe PEI-based antifungal nanovaccine. The antigen consists of the protein Heat shock protein 90-CTD (Hsp90-CTD), which is a breakdown product of the fungal protein Hsp90. It was reported that Hsp90 would be a protective antigen following the detection of high levels of Hsp90-specific antibodies in sera of patients who recovered from systemic candidiasis (16–18). Studies showed that patients who recover from systemic candidiasis produce a major antibody response to Hsp90-CTD protein, whereas fatal cases have little antibody or falling titers (8, 10). However, the immunogenicity of antigen is too weak to elicit immune responses strongly (19). In this study, we introduced PEI as the adjuvant of the nanovaccine. PEI was incorporated into the NP structure to form nanovaccine by combining positive and negative charges with the antigen as an adjuvant, which enhanced the immune response *in vivo* of mice. The results showed that the prepared antifungal nanovaccine could induce a rapid immune response in mice and provide long-term antibodies *in vivo* by inducing B cells to differentiate into long-lived plasma cells.

## Material and Methods

### Materials

Polyethylenimine (PEI) (branched form, molecular weight 25 kDa) was obtained from Sigma-Aldrich (US). Sodium dodecyl sulfate (SDS) was purchased from Aladdin Chemistry Co. Ltd (Shanghai, China).

### Mice

C57BL/6 mice were obtained from the Shanghai Laboratory Animal Center. All experiments on animals were conducted in strict compliance with the Regulations for the Administration of Affairs Concerning Experimental Animals approved by the State Council of People’s Republic of China. The protocol used was approved by the Institutional Animal Care and Use Committee of Tongji University (Permit Number: TJAA08021101).

### Expression and Purification of Recombinant His-Tagged Hsp90-CTD

Protein Hsp90-CTD was established by total gene synthesis by Tsingke Biotechnology (Shanghai, China), and its expression plasmid was cloned into the pET21a vectors (Novagen). Recombinant plasmids were transformed into *Escherichia coli* BL21 (DE3)-RIPL cells (Takara Biotechnology Company, Beijing, China), which were grown in Luria-Bertani (LB, 1.0% tryptone, 0.5% yeast extract, 1.0% NaCl, and 1.5% agar) plates medium containing ampicillin. We isolated the individual clone and cultured in liquid LB medium containing ampicillin for 16 h at 16°C. The bacterial solution mixture was centrifuged at 8000 rpm for 3 min, and the pellet was then resuspended in phosphate-buffered saline (PBS) and collected. The bacterial suspension was sonicated for 3 circulations of 180 s (300 W) at 0°C, protein was collected. Hsp90-CTD-His6 was purified *via* a one-step Ni-affinity column using washing buffer and elution buffer for the purification. The existence and purity of recombinant protein was evaluated by SDS-PAGE.

### Fungal Strain and Cell Line

*C. albicans* SC5314 was kindly provided by Sanglard D (Centre Hospitalier Universitaire Vaudois). All fungal strains were routinely cultured on sabouraud dextrose agar (SDA) plates (1% peptone, 4% dextrose, and 1.8% agar) for isolation of individual clones and cultured in yeast peptone dextrose (YPD) liquid medium (1% yeast extract, 2% peptone, and 2% dextrose) at 30°C in a shaking incubator.

RAW264.7 macrophages, provided by Cell Resources Center of the Chinese Academy of Sciences, were cultured in DMEM medium supplemented with 10% (vol/vol) heat-inactivated fetal calf serum (FCS) at 37°C with 5% CO_2_.

### Three-Dimensional (3D) Structure Modeling and Docking of Protein and SDS

Entered protein sequence in SWISS-MODEL (http://swissmodel.expasy.org/) and model 3D protein Hsp90-CTD structure.

Based on the highest sequence coverage and identity (>30%), the best template was selected for modeling each Hsp90-CTD. The 2D structure of SDS was taken from PubChem (https://pubchem.ncbi.nlm.nih.gov). The energy was minimized using Chem 3D Pro and converted to MOL 3D structures using Open babel and saved as MOL2 format. For the prediction, SDS was selected as ligand for docking with protein Hsp90-CTD as the receptor. Binding sites of the proteins were identified using Discovery Studio, and structure visualization was carried out with PyMol. It predicted that SDS can bind to protein.

### SDS “Charged” Hsp90-CTD

**Hsp90-CTD plus different doses of SDS.** We used a zeta potential analyzer (Zetasizer Nano ZS, ZEN 3690, Malvern) to measure the surface potential of Hsp90-CTD. We explored the changes in the charged amount of the protein after SDS binds to Hsp90-CTD. Native-PAGE (30% Acr-Bis, TEMED, 1M Tris-HCl pH8.8 or pH6.8) was performed with a 10% acrylamide gel without SDS, and electrophoresis solutions contained no SDS or denaturing agents. As mobility in native gels is dependent on both protein size and charge (20), the presence of a lower band on the PAGE gel could be attributed to differences in charge between Hsp90-CTD plus different doses of SDS.

### Preparation of NP@PEI+Hsp90-CTD

The concentration of protein Hsp90-CTD was diluted to 0.5 mg/ml, and 1 ml-protein was added in a centrifuge tube. It was then added to the 1/5 volume of SDS (0.1 mg/ml) sample buffer and sonicated for 5 min to combine the two substances. We added PEI (0.5 mg/ml, W_PEI_: W_Hsp90-CTD_=1:7) solution to the aforementioned mixture, and sonicated again for 5 min to prepare nanoparticles (NP@PEI+Hsp90-CTD). Finally, the NP was subject to characterization.

### Characterization of NP@PEI+Hsp90-CTD

The particle size and distribution of PEI+Hsp90-CTD complex and NP@PEI+Hsp90-CTD were determined by dynamic light scattering (DLS) (Zetasizer Nano ZS, ZEN 3690, Malvern). The morphology of nanoparticle was observed by TEM (Tecnai-12Bio-Twin, FEI, Netherlands). The conformation of Hsp90-CTD was investigated using circular dichroism (CD) spectrometer (J-810, JASCON CO, LTD, Japan).

### Cytotoxicity Evaluation of NP@PEI+Hsp90-CTD

Cytotoxicity was assayed using CellTiter-Lumi (CTL, Beyotime, Shanghai, China). RAW264.7 macrophages were seeded in 96-well cell culture plates (2 × 10^4^ cells/well) and treated with NP@PEI+Hsp90-CTD, Hsp90-CTD, PEI, and SDS for 4.5 h. The cytotoxicity of particles was assayed using a cell viability assay kit (Beyotime) according to the manufacturer’s instructions. There was 100 μL of CTL solution added to each well (100 μL complete culture medium), and the concentration of CTL used is 50%. Cell viability was measured as luminous intensity at 450 nm, each group had three repeats.

Cytotoxicity was assayed using Cell Counting Kit-8 (CCK8, Beyotime, Shanghai, China). RAW264.7 macrophages were seeded in 96-well cell culture plates (2 × 10^4^ cells/well) and treated with NP@PEI+Hsp90-CTD, Hsp90-CTD, PEI, and SDS for 6 h. Then 10 μL of CCK8 solution was added to each well (100 μL complete culture medium), followed by 1 h of incubation at 37°C. Cell viability was measured as luminous intensity at 500 nm, and each group was repeated three times.

### Serum Antibody Titer Measurement

Indirect enzyme-linked immunosorbent assay (ELISA) was used to determine the antibody titer in the sera of animals. In brief, protein Hsp90-CTD (1 µg/ml) was coated into 96-well ELISA plates at 4°C overnight. After blocking with 5% bovine serum albumin (BSA), washing in PBS supplemented with 0.05% Tween 20 (PBST), different group dilutions of immune sera (1/500 in PBS) were added and incubated at 37°C overnight. After washing in PBST, horse-radish peroxidase (HRP)-conjugated sheep anti-mouse IgG secondary antibody (1:10000, NA931, GE Healthcare Life Sciences) was added and incubated at 37°C for 1 h. After washing in PBST, 100 μL of TMB (3, 3’, 5,5’-Tetramethylbenzidine) substrate solution was added to each well and incubated for 7 min at room temperature. Reactions were then stopped by the addition of 2M H_2_SO_4_. Optical absorbance (OD450/490 nm) was read in a microplate reader (Molecular Device).

### Evaluation of Long-Lived Plasma Cells Response by Flow Cytometry

For flow cytometry analysis of long-lived plasma cells (LLPCs), bone marrow (BM) cells were isolated and stained with different antibodies. The BM cells were flushed with RPMI-1640 from the femurs and tibiae of immunized mice. Erythrocytes were removed using the red blood cell lysis buffer, and bone marrow cells were cleaned and resuspended with FACS (5% fetal bovine serum +PBS). Cell suspensions from mouse bone marrow were stained using the following fluorescent-labeled anti-mouse antibodies: Anti-CD45/B220, APC anti-CD138, PE anti-CD44, and FITC anti-MHCII. Following a 30 min incubation at 4°C, cells were washed in PBS and fixed with Fix Buffer (4% paraformaldehyde: FACS=1:3) overnight at 4°C. Data were acquired on FACS Canto (BD FACSVerse) flow cytometer and analyzed using FlowJo (version 10, https://www.flowjo.com) software.

### Adoptive Transfer

Ten C57BL/6 female mice were randomly divided into two groups (5/group). Mice in the immunized group were treated with nanovaccine two times at an interval of 14 days. Immunization sera were collected on Day 28. Mice in the blank group were sacrificed and sera were collected on the same day. Both of two sera were injected into another 6 mice (180 μL per mouse) through the tail vein (3/group). After 1 h, mice were injected with 200 μL of a suspension containing live *C. albicans* SC5314 (1 × 10^6^ CFU/LD50) in sterile saline *via* the lateral tail vein. At 48 h post-infection, the mice were sacrificed and the kidneys were removed, and then fixed in 10% neutral formalin for Hematoxylin-eosin Staining (H&E) and Periodic Acid-Schiff Stain (PAS) staining, respectively.

### Statistical Analysis

All data were compared using t test, paired t test, and one-way ANOVA. Statistical significance was set at one of the following p-values in the figures as: *P < 0.05; **P < 0.01; ***P <0.001; ns, P>0.05. Software Graphpad prism 7 was used to evaluate data.

## Results

### Expression of Recombinant Protein Hsp90-CTD

**Figure 1:**
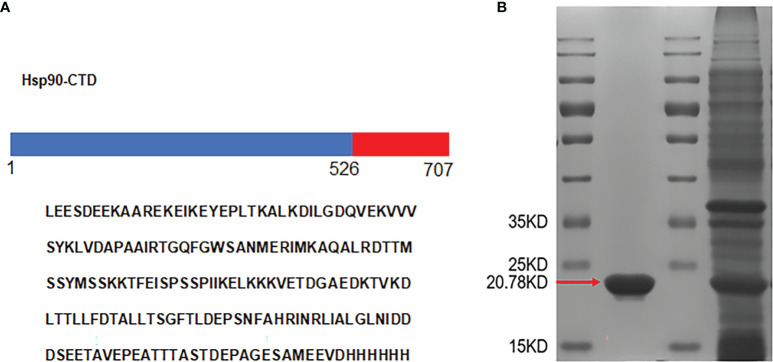
Design and expression of antigen. **(A)** The amino acid sequences of the recombinant protein Hsp90-CTD. **(B)** The His-tagged recombinant proteins were purified by Ni-affinity chromatography and analyzed by SDS-PAGE. Red arrow shows monomers of Hsp90-CTD. The other band was from the supernatant that contained soluble proteins.

Hsp90 protein has a total length of 707 amino acids, of which the C-terminal (Hsp90-CTD) can produce effective antifungal antibodies. Hsp90-CTD plasmid was constructed with 181 amino acids. The recombinant protein Hsp90-CTD had 6 His-tags at the sequence end, which facilitated their purification over an Ni/NTA column (**Figure 1A**). Expression of Hsp90-CTD protein was performed in *E. coli* BL21 (DE3) strain and 800 ml of bacterial broth was harvested. The protein solution was collected after ultrasonic lysis of the bacterial broth and purified on an Ni/NTA column. The concentration of the target protein obtained after purification was 2.1 mg/ml in a volume of 5 ml, and the total amount was 10.5 mg. The expression of the protein was predicted molecular weights of 20.78 kDa and confirmed by the band size in the SDS-PAGE analysis (**Figure 1B**).

### Synthesis and Characterization of Hsp90-CTD Nanovaccine

**Figure 2:**
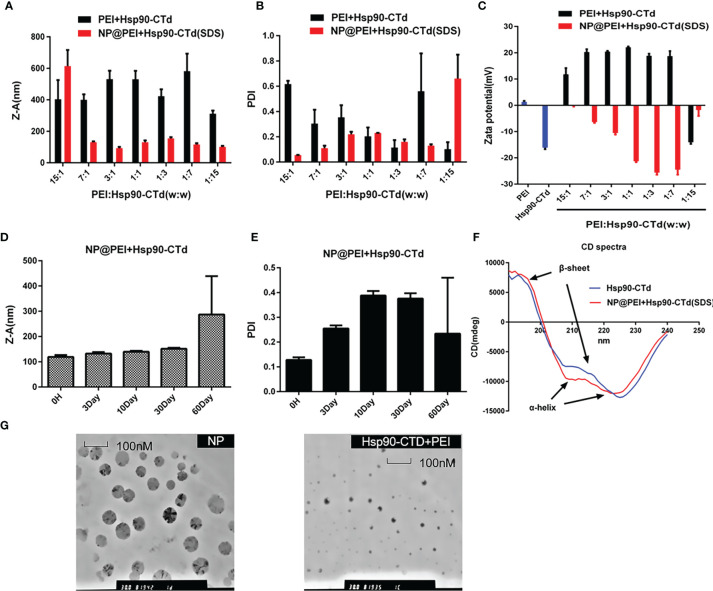
Synthesis and characterization of the particles. **(A)** Size of different proportions (PEI: Hsp90-CTD) nanoparticles after treating with SDS. **(B)** PDI of NP@PEI+Hsp90-CTD nanoparticles after treating with SDS. **(C)** Surface zeta potential of nanoparticles. The nanoparticles’ stability of **(D)** size, and **(E)** PDI stored at 4°C for a month. **(F)** Comparable results between pure protein and SDS-modified protein were obtained by circular dichroism (CD) spectroscopy. **(G)** TEM images of NP@PEI+Hsp90-CTD and particles without SDS (Hsp90-CTD+PEI).

As shown in **Figures 2A, B**, PEI and Hsp90-CTD are combined in different proportions according to weight. It was found that after adding SDS, the size and PDI value of the synthesized particles was smaller and more stable. The purpose of introducing SDS is to add a negative charge to the protein (21, 22) so that it can bind more easily to PEI to form nanoparticles. After comparison, W_PEI_ : W_Hsp90-CTD_=1:7 was the best ratio. The size distribution of NP@PEI+Hsp90-CTD (NP) was observed by DLS, the NP had an average size of 116.2 nm with a PDI of 0.13 (**Figures 2A, B**). According to **Figure 2C**, the untreated PEI had a positive charge (+1.28mV), the untreated Hsp90-CTD had a negative charge (-16.1 mV), and the potential of NP (with SDS) had a negative changed significantly. When SDS acts on the hydrophobic region of the protein, it can promote the formation of nanoparticles between PEI and protein more easily. In **Figure 2D**, the size of NP was below 200 nm in 30 days. In **Figure 2E**, the polydispersity index (PDI) value of NP indicated that these nanoparticles showed stability within a month. Conformational changes of Hsp90-CTD upon interaction with SDS and PEI were monitored using circular dichroism (CD) (**Figure 2F**). Compared with protein Hsp90-CTD, the α-helix and β-sheet of antigen protein which formed the nanovaccine were changed, respectively. In **Figure 2G**, typical surface morphologies of the nanovaccine were observed by TEM, nanoparticles had more uniform size and more regular spherical structure than particles with no SDS.

### SDS Increased the Negative Charge of Antigen and Promote the Formation of Nanoparticles With PEI

**Figure 3:**
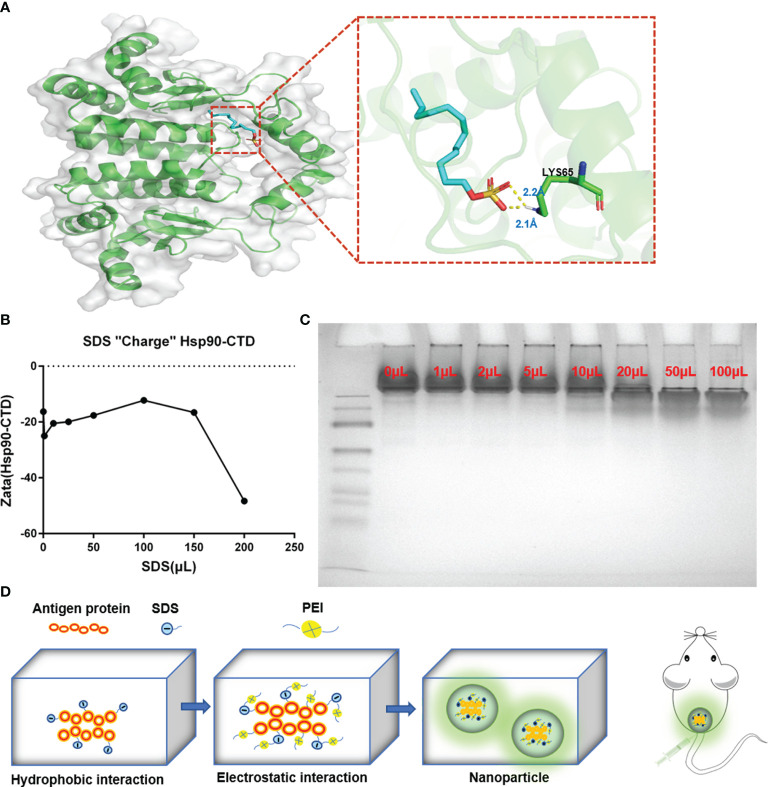
Formation mechanism of Hsp90-CTD nanovaccine. **(A)** The putative binding site of SDS on protein Hsp90-CTD as obtained from Autodock, shows the hydrophobic interaction between SDS and Hsp90-CTD. Molecule SDS forms 2 hydrogen bonds with amino acid LYS65, acting at distances of 2.3 Å and 2.1 Å **(B)** DLS detects the solution potential value. Changes in potential of Hsp90-CTD protein combined with different volumes of SDS solution. **(C)** Native-PAGE: The migration efficiency of protein Hsp90-CTD combined with different volumes of SDS solution was analyzed on 10% Native-PAGE. **(D)** Schematic illustration of the process for synthesizing nanoparticles. Hsp90-CTD and SDS were combined with hydrophobic force. Then the polymer PEI was introduced to combine with the mixture by electrostatic force. Finally, well-characterized nanoparticles were formed.

Sodium dodecyl sulfate (SDS) is a surfactant with a 12-carbon tail attached to a sulfate group, according to its negative polar head group, also called anionic surfactant (23, 24). There are generally two modes of SDS-protein binding. The majority of binding is hydrophobic binding, which is a non-specific weak interaction. The other binding is a specific strong interaction (hydrophilic binding) (25). The molecular docking method was employed using the AutoDock to predict the binding site for SDS on protein Hsp90-CTD (**Figure 3A**). Molecule SDS forms 2 hydrogen bonds with amino acid LYS65, acting at distances of 2.3 Å and 2.1 Å. The tail of the SDS can form a complex with the protein in the hydrophobic region through the hydrophobic interaction, while the negative polar head group is exposed in the hydrophilic region (21, 26). The process not only increases the hydrophilicity of the protein but also increases the negative charge of the protein load (21, 27). To confirm this point, different volumes of SDS solution (0.1 mg/mL) were added to the quantitative Hsp90-CTD solution (0.8 mL, 0.1 mg/mL) to measure the potential. The negative charge of protein Hsp90-CTD increased with the increase of SDS (**Figure 3B**). Native PAGE also proves the view that combined with higher doses of SDS, the protein can travel farther from the negative pole to the positive pole through the gel (**Figure 3C**). As shown in **Figure 3D**, dissolve the protein Hsp90-CTD (0.5mg/mL) in ultra-pure water, sonicating while adding SDS (1mg/mL). After 5 min, add PEI (0.5 mg/mL), the particles in the system to form nanoparticles *via* positive and negative electricity. Size and PDI were measured to optimize the ratio of Hsp90-CTD : PEI weight in nanoparticles preparation. SDS can increase the negative charge of antigen and promote the formation of nanoparticles with PEI.

### PEI-Based Nanovaccine Induces A Rapid Immune Response

**Figure 4:**
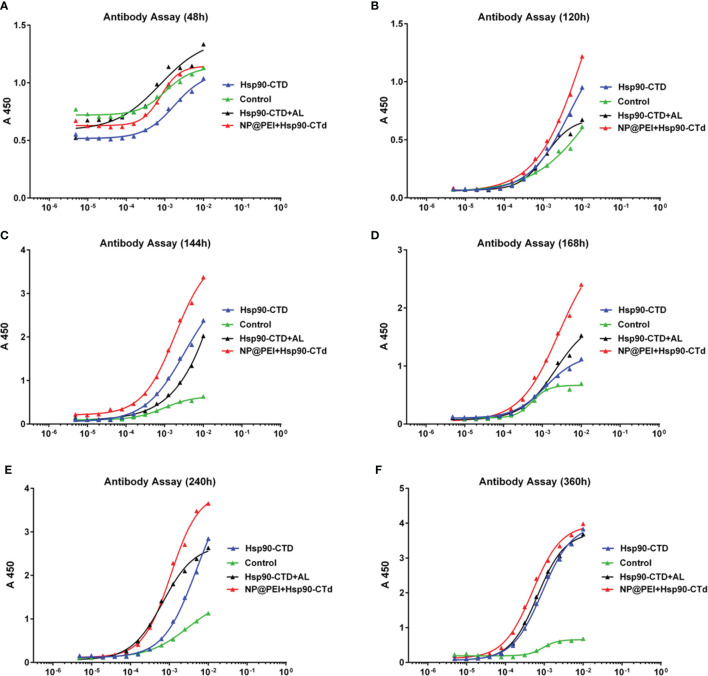
Hsp90-CTD (1 µg/ml) was coated into 96-well ELISA plates at 4°C overnight. Blood from 4 groups (control, Hsp90-CTD, Hsp90-CTD+AL, and NP) of mice was collected at different time points, 3 mice/group. Sera were obtained after centrifugation at 9000 rpm for 10 min. The sera were diluted 500 times and the antibody levels were then measured by ELISA. **(A)** 48 h **(B)** 120 h **(C)** 144 h **(D)** 168 h **(E)** 240 h **(F)** 360 h.

Once uptaking presentable antigens, immature dendritic cells (DCs) turn to mature DCs with capability of antigen-presenting capability. PEI-based antigens were recognized by mature DCs and rapidly stimulated immune cells, which then produced antibodies. In this study, ELISA was used to detect the antibody titer in the serum of mice which were immunized. The results showed that nanovaccine can stimulate the maturation of bone marrow derived cells (BMDC) and stimulate the immune response to produce antibodies more quickly (**Figure 4**). There were12 mice (C57BL/6) randomly divided into 4 groups with 3 mice in each group. Groups of mice were injected subcutaneously at the tail base (protein, nanovaccine, and protein+AL adjuvant) with the same dose of antigen protein Hsp90-CTD 10 μg. We evaluated the production of high affinity antibodies by performing ELISA with serum of mice bled 48 h, 120 h, 144 h, 168 h, 240 h, and 360 h after immunization. First, blood was drawn from the eye canthus to obtain serum and serum was separated by 9000 rpm centrifugation. Then the serum was diluted 100 times with PBS and tested. The ELISA results showed that there was little difference in the antibody titers at 24 h (**Figure 4A**). In addition, 120 h after immunization, the antibody titer of the NP-inject group was a little higher than the other groups (**Figure 4B**). After 144 h, 168 h, and 240 h, we found that the antibody titer of the NP-inject group reached a higher level significantly faster compared to other groups (**Figures 4C–E**). Until 360 h, the antibody titer tended to be the same in all groups (**Figure 4F**).

### PEI-Based Nanovaccine Induces Long-Term Secretion of Antibody

**Figure 5:**
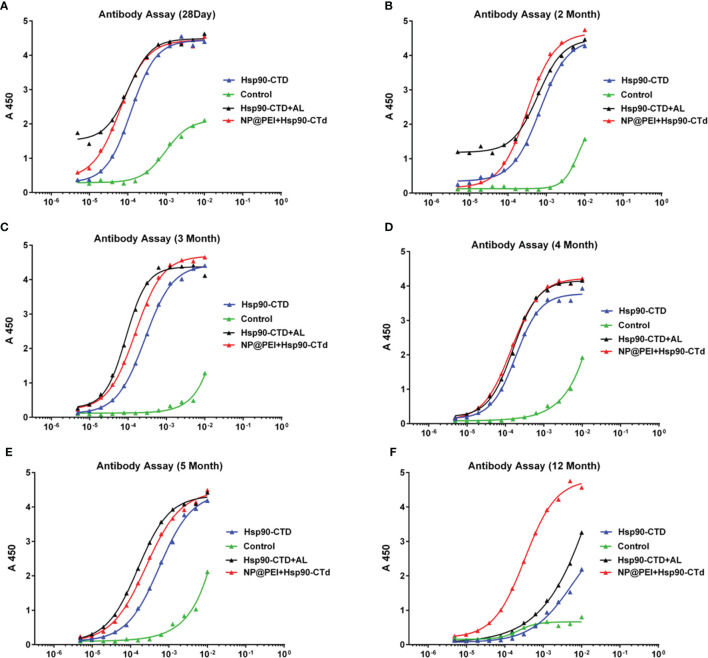
Hsp90-CTD (1 µg/ml) was coated into 96-well ELISA plates at 4°C overnight. Blood from 4 groups (control, Hsp90-CTD, Hsp90-CTD+AL, and NP) of mice was collected at different time points, 3 mice/group. Sera were obtained after centrifugation at 9000 rpm for 10 min. The sera were diluted 500 times and the antibody levels were then measured by ELISA. **(A)** 28 days. **(B)** 2 months. **(C)** 3 months. **(D)** 4 months. **(E)** 5 months. **(F)** 12 months.

It is a great challenge when vaccine efficacy requires high serum antibody titers, combined with long-lived antibody responses (28). To study whether the antibody of immunized mice can exist for a long time, we determined antibody titers *in vivo* at different stages. There were12 mice (C57BL/6) randomly divided into 4 groups with 3 mice in each group. Groups of mice were injected subcutaneously at the tail base (protein, nanovaccine, and protein+AL adjuvant) with the same dose of antigen protein Hsp90-CTD 50 μg. The antibody titers were determined *via* ELISA. The results showed that the antibody titers of different groups were very close at the points within 5 months (**Figures 5A–E**), until 12 months. At the point of 12 months, the antibody titers of the NP group still maintained a high level, which was significantly ahead of other groups (**Figure 5F**). Adjuvant PEI of nanovaccine helps to obtain long-term antibodies in the body, then we try to find out the mechanism.

### Nanovaccine Can Promote Differentiation of B Cells to Long-Lived Plasma Cells

**Figure 6:**
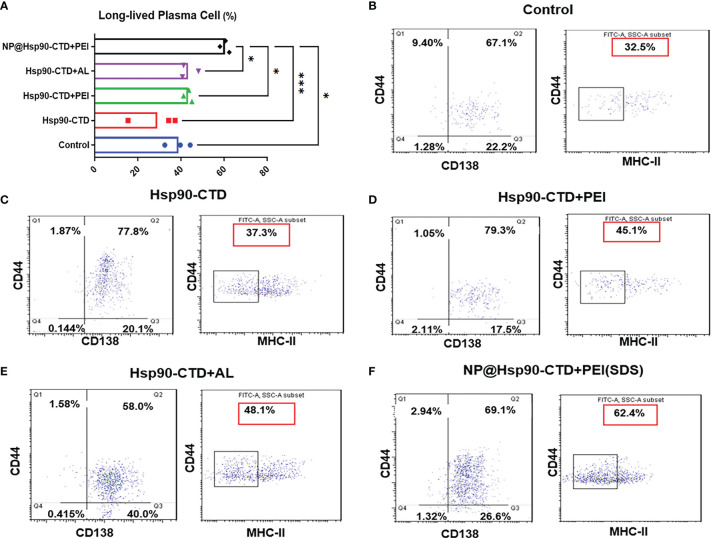
Levels of LLPCs in each group of mice detected by flow cytometry. CD44/CD138 gating signify plasma cells, and CD44/MHC II gating signify long-lived plasma cells. 1*104 cells per condition were analyzed by flow cytometry. The proportion in the red box represents the proportion of LLPCs. **(A)** LLPCs of different groups mice. The proportion of LLPCs in the NP@Hsp90-CTD+PEI group was significantly higher than that in other groups, especially in the Hsp90-CTD group. *P < 0.05; ***P < 0 .001. **(B)** LLPCs of group control mice. **(C)** LLPCs of group pure protein mice. **(D)** LLPCs of group Hsp90-CTD+PEI mice. **(E)** LLPCs of group Al adjuvants mice. **(F)** LLPCs of group NP mice.

Studies have shown that long-lived plasma cells (LLPCs) are differentiated from B cells in germinal centers (GCs) and are largely responsible for the long-term secretion of antibodies (29–31). GCs are specialized structures in B cell follicles of secondary lymphoid tissues where somatic hypermutation of high-affinity B cells occur, followed by differentiation of LLPCs or memory B cells (32, 33). Newly generated LLPCs then migrate mainly to the bone marrow and memory B cells remain quiescent (34). Memory B cells reactivation requires re-encountering the same antigen, but the long-lived plasma cells can sustain levels of antibodies during periods of little or no exposure to infection (35, 36). Since mice still have high titers of antibodies after one year of immunization, it is speculated that the number of LLPCs in the mice increased after NP immunization, which could maintain long-term antibodies in the body. To investigate the LLPCs in immunized mice, flow cytometry (FCM) was performed with bone-marrow cells. Fifteen C57BL/6 female mice were randomly divided into 5 groups (3/group). Mice were inoculated subcutaneously (50 μg Hsp90-CTD per mouse) with the following 4 formulations: pure Hsp90-CTD, Hsp90-CTD+PEI, Hsp90-CTD+AL, and NP@Hsp90-CTD+PEI. Mice were immunized two times at an interval of 14 days. Then, we evaluated the production of long-lived plasma cells in mice that were boosted with nanovaccine 28 days after immunization. Mice were sacrificed, the bone marrow cells of mice were collected. We stained LLPCs with B220^hi^, CD44^hi^, CD138^hi^, and MHCII^lo^. **Figure 6A** shows the average number of LLPCs in each group for comparison, we can see that LLPCs of the NP group were significantly higher than other groups. **Figure 6B** shows the percentage of LLPCs from control mice. The LLPCs of mice of group pure Hsp90-CTD, group Hsp90-CTD+PEI, group Hsp90-CTD+Al, and group NP were shown in **Figures 6C–F**. The results suggested that nanovaccine can cause differentiation of B cells to LLPCs, and induce long-lasting immunological protection.

### The Serum of Immunized Mice Has a Protective Effect on Infected Mice

**Figure 7:**
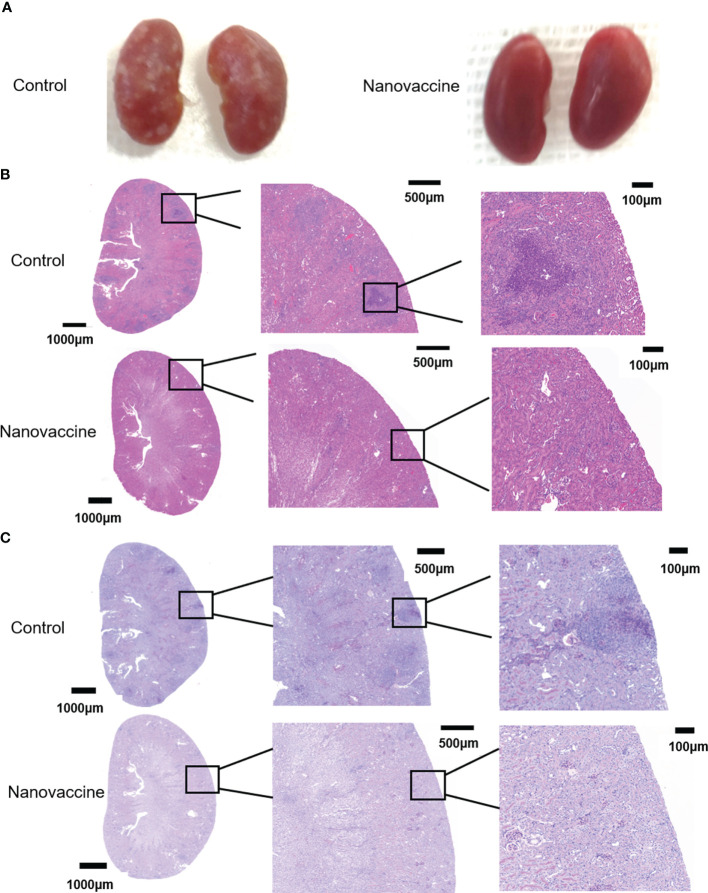
The infected kidneys were protected by the serum of nanovaccine-immunized mice. **(A)** Kidneys of mice were protected with serum of the control group and the nanovaccine group, respectively. The kidneys of unprotected mice were infested with *C. albicans* SC5314 and the surface was already covered with it. In contrast, the kidneys of antibody-protected mice had a very smooth surface. **(B)** Representative H&E staining of kidneys from infected mice with indicated treatment at 48 h post-infection. **(C)** Representative PAS staining of kidneys from infected mice with indicated treatment at 48 h post-infection. The darker areas framed by red lines were the locations of fungal infestation inside the kidney.

To evaluate the protective effect of the antibody on *C. albicans* virulence in C57BL/6 mice, we established a fungal infection model and treated infected mice with sera from immunized mice. C57BL/6 mice could accumulate high fungal loads within 72 h. Kidney damage is a common feature of *C. albicans* infection (37). Mice in the immunized group were treated with nanovaccine two times at an interval of 14 days. Immunization sera were collected on Day 28. Five model mice were injected with PBS as control and sera were collected on the same day. Immunized sera and blank sera were adoptively transferred *via* the tail vein into two groups (3 mouse/group) of blank C57BL/6 mice (180 μL/mouse). These animals were challenged with *C. albicans* SC5314 (1×10^6^/mouse) 1 h after receiving the sera. *C. albicans* was intravenously injected into the mice, followed by the analyses of host kidneys 3 days after infection. Mice were sacrificed and kidneys were taken. The perirenal membrane of the kidney was removed, then its surface was photographed and recorded. It can be clearly seen that the kidney surface of the control group has been attacked by the fungus. The serum-protected kidney had no obvious infection, and its surface was very smooth (**Figure 7A**). To investigate the internal infection of the kidneys by the fungus, they were treated with H&E-stain and PAS-stain. The kidneys were fixed, embedded in paraffin, and sectioned. The pictures showed that the interior of the kidney, which is not protected by antibodies, had been attacked by the fungus. We can see immunized sera treatment decreases kidney tissue damage in mice, while blank sera cannot provide protection (**Figures 7B, C**).

### Low Toxicity of PEI-Nanovaccine

**Figure 8:**
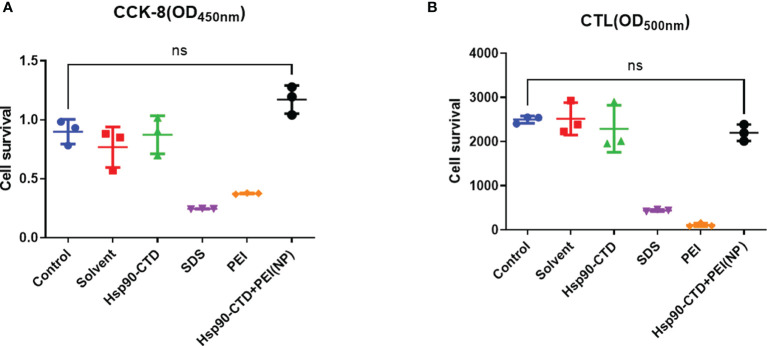
Detection of nanoparticle toxicity. **(A)** Cell survival was measured with CCK8 assay after nanovaccine injected. **(B)** Cell survival was measured with CTL assay after nanovaccine injected. No significant difference between the experimental group and the control group. P > 0.05.

Before injecting, this study investigated the cytotoxic effect of 6 groups: control, solvent, pure protein, SDS solution, PEI solution, and NP by cell survival assays. This study measured the cell viability of 6 groups: control, solvent, pure protein, SDS solution, PEI solution, and NP. We measured the cell viability of particles *in vitro* by CCK8 assay and CLT assay (**Figures 8A, B**). We treated the cells with the above six different solutions for 6 h, and the results showed that the solution of SDS and PEI had obvious cytotoxic effects. **Figures 8A, B** also indicate that nanoparticle treatment at these concentrations has no cytotoxic effect on cells compared with the control group.

## Discussion

*C. albicans* is one of the most common reasons of Candida infections, also causing high mortality and morbidity in immunocompromised patients (38). A mortality of 40% has been reported in patients with invasive candidiasis, even with the proper treatment with current antifungal drugs (39). Antifungal vaccine is the primary method of prevention, especially for those with weakened immune systems. However, with the increase in immunocompromised patients, the clinical need for fungal vaccines to prevent these deadly infections has not been met. With the further study of host-fungus interactions, significant progress has been made in the research of antifungal vaccines. In recent years, research about antifungal vaccines has become more popular, and the immunogenicity and efficacy of vaccines have been confirmed in animal models. Over the years, numerous groups have developed a variety of candidate antifungal vaccines against *C. albicans*, and evaluated the bactericidal and long-term antifungal immunity of the vaccines. The antigens of these vaccine candidates include fungal protein subunits, fungal cell-wall polysaccharide, and live attenuated fungi (6). Agglutinin-like sequence (Als) proteins locate to the surface of *C. albicans*, which is one of the most promising vaccine strategies ever tested (40, 41). Xin et al. developed an innovative β-mannan vaccine, which can induce significant protection against experimental disseminated candidiasis in mice (39, 42). In addition, live attenuated strains of *C. albicans* have also been explored in mouse models (43, 44) (40, 41), they have not been tested in humans because of the high risk to humans. The secreted aspartyl proteinase (SAP) family has been described as a major virulence factor in *C. albicans*. Naglik et al. (45) analyzed gene expression in more than 130 subjects with *C. albicans* infections and found that SAP2 and SAP5 were the most commonly expressed genes. According to Edwards’ review article (46), the Sap2p vaccine is one of the vaccine candidates and has already passed a Phase I clinical trial against the infection. The cell wall proteins obtained through β-mercaptoethanol (β-ME) extraction of *C. albicans* can also be used as antigens in the preparation of vaccines. Thomas’ study found that β-ME extract could provide protection against fungal infection in mice (47). These antifungal vaccines with different types of antigens have all made varying degrees of progress. No studies have been conducted on the production of long-term antibodies to antifungal vaccines *in vivo*. Recently, Freund’s adjuvant has been extensively used in animals, especially in mice models. But now it is no longer recommended because of its painful reaction and potential tissue damage. Instead, the alum adjuvant is widely used in antifungal vaccine development. However, we did not find reports that a certain adjuvant could elicit long-term antifungal antibodies to the vaccine. In this study, PEI as adjuvant while Hsp90-CTD as antigen were prepared in nanovaccine and explored the long-term antibody *in vivo*.

In the process of preparing the nanovaccine, we found that PEI was not able to form nanoparticles of good quality when combined with antigenic proteins alone. Therefore, we introduced SDS into the system and made it easier for the protein and PEI to form smaller and more uniform nanoparticles. The reason is that as an anionic surfactant, the tail of SDS can bind to the hydrophobic region of protein Hsp90-CTD in a hydrophobic manner. The negative charge of the compound is increased significantly, and the compound can combine with a positive charge PEI to form nanoparticles. We demonstrated that the antigen protein Hsp90-CTD can bind to SDS and its negative charge is increased. Then add PEI, we got a nanovaccine and the average size of particles with or without SDS was determined. SDS acts as a binder, making it easier for proteins Hsp90-CTD and PEI to form nanoparticles. Circular dichroism showed some changes in the structure of the protein as it forms nanoparticles. The α-helical structure shows the orderliness of the protein structure, while the β-fold shows the looseness of the protein. The antigen protein shows negative peaks at 208 nm and 222 nm, and the peaks are displaced after the formation of nanoparticles, representing enhanced hydrophilicity. An increase in the proportion of β-folding represents a decrease in the structural order of the protein molecule. We are working to develop an antifungal vaccine with long-term effects. C57BL/6 mice were used to establish an animal model. Regarding the study of fungal vaccines, most investigators consider C57BL/6 as a vaccine model mouse to be suitable (48–50). C57BL/6 mice are characterized by stability and a tendency to elicit immune responses. Groups of mice were injected subcutaneously (51–53) at the tail base (protein, nanovaccine, and protein+AL adjuvant) with the same dose of antigen protein Hsp90-CTD 50 μg. Then, we detected the maintenance time of antibody in mice after immunization. We found that the antibody titers were still high in the mice that received the nanovaccine after 12 months (**Figure 5**). Hsp90-CTD can produce antibodies when used as an antigen alone, but our studies have shown that without PEI as an adjuvant to assist, the antibody titers *in vivo* do not maintain high levels over time. The results showed that the PEI-based nanovaccine can cause the presence of long-term antibodies in mice.

Studies have shown that long-lived plasma cells are important plasma cells that can maintain long-term antibodies in the body (29–31). Therefore, we hope to be able to detect LLPCs in vaccine-immunized mice. After antigenic stimulation, naive B cells undergo extensive proliferation in the follicles forming the GC in lymphoid tissues (54). GCs are dynamic microenvironments for B cell differentiation after antigen stimulation (55). B cells respond quickly, and some of the B cells differentiate into LLPCs (56). LLPCs then move to the BM, where they reside and produce antibodies for extended periods (57). Nanovaccine was injected subcutaneously into C57BL/6 mice and bone marrow cells were collected 30 days later for flow cytometry. LLPCs were analyzed by a flow analyzer, and we found that the proportion of LLPCs in BM is significantly higher compared to other forms of antigens. After being ingested by BMDC, nanovaccine was presented to immune cells. PEI as adjuvant not only promoted the rapid production of antibody *in vivo*, but also promoted the differentiation of B cells into LLPCs, so as to maintain the long-term existence of antibody *in vivo.* Therefore, we concluded that PEI-based nanovaccine is the key to enable the production of long-term antibodies *in vivo*. It is also important that antibodies are “effective” antibodies, so we tested the protective effect of sera containing antibodies for fungal-infection mice from immunized mice. We established a *C. albicans* infected mouse model and treated it with serum from immunized mice. The H&E and PAS staining results showed that serum from immunized mice had a protective effect on the kidneys of fungal-infected mice compared with serum from control mice. We can clearly see in **Figure 7** that the serum of non-immunized mice has almost no protective effect on the kidney of mice, while the serum of immunized mice reduces the damage of *C. albicans* to the kidney.

**Figure 9:**
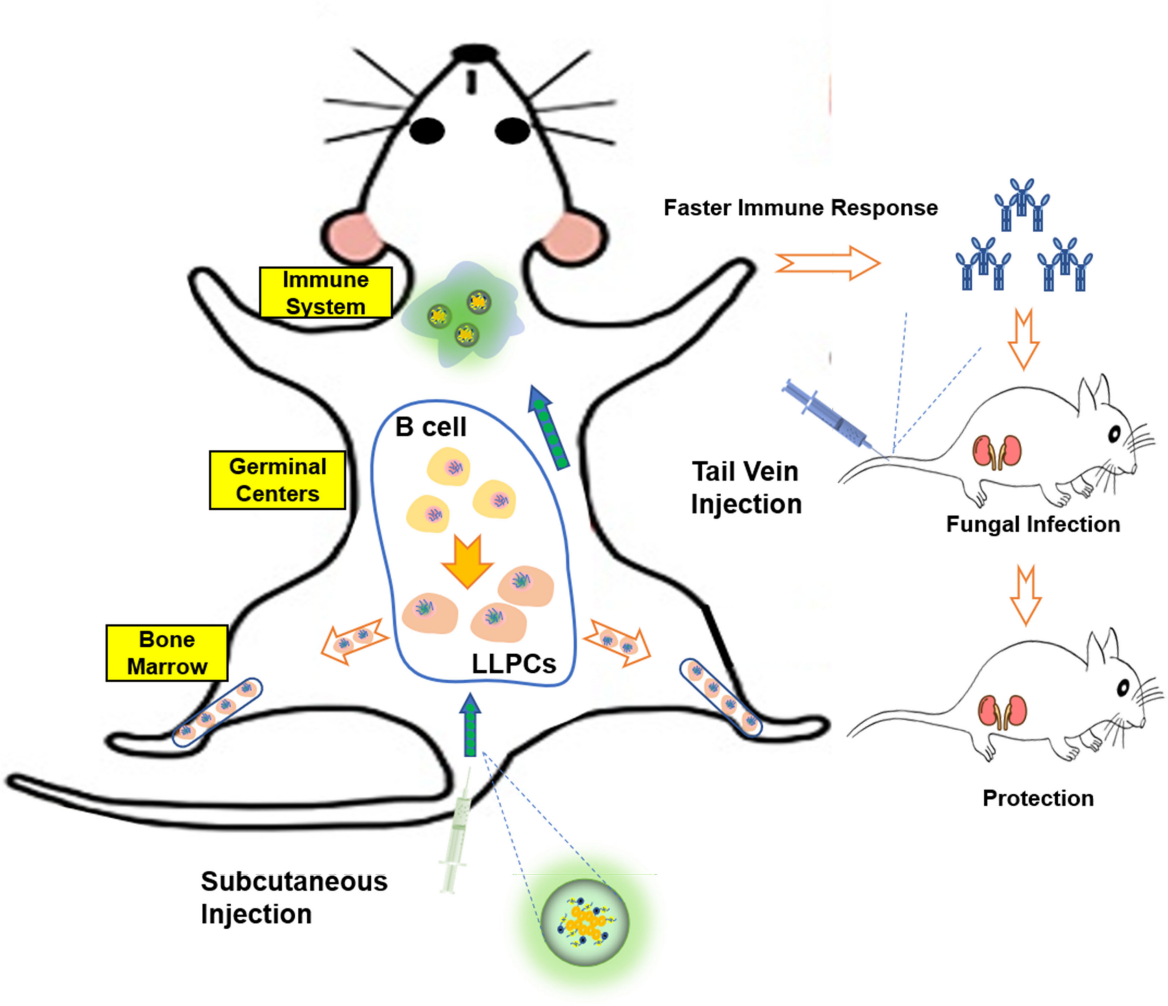
Schematic representation of Hsp90-based antigen delivery system and its role in eliciting cellular immunity response. Nanovaccine in mice caused immune response, so that the body quickly produces antibodies and plays a protective role. At the same time, the vaccine can promote the differentiation of B cells into LLPCs, so that the body has long-time protective antibodies.

Collectively, this study demonstrated that PEI-based nanovaccine can promote antigen presentation to mature B cells in GC, and facilitate differentiation of B cells into long-lived plasma cells that populate the bone marrow. NP not only can induce faster antibody titer but also can cause long-term antibodies *in vivo* (**Figure 9**). Although at this stage of our research we can only provide some ideas, we have laid a solid foundation for future research on long-acting vaccine.

## Conclusion

In recent years, the robust adjuvanticity of PEI has been continuously explored and verified. Research has shown that PEI-based particles can improve the efficiency of conventional vaccines against tumors. Based on previous studies, this study expanded the use and function of PEI as adjuvant. We linked PEI with antifungal antigen protein Hsp90-CTD to form a nanovaccine and found that PEI-based nanovaccine not only can make mice produce antibodies quickly, but also make mice have protective antibodies for a long time. The present design increases the sustained effect of antifungal vaccine, and provides a new strategy for vaccines in the field of long-term vaccine research.

## Data Availability Statement

The original contributions presented in the study are included in the article/supplementary material. Further inquiries can be directed to the corresponding authors.

## Ethics Statement

The animal study was reviewed and approved by The Ethics Committee of Tongji Medical College.

## Author Contributions

ZJ designed the experiments under the mentorship of HS, M-MA, and Y-YL. ZJ, X-RQ, Y-TD, S-MC, and YZ performed the sequence design and expression of antigenic proteins under the mentorship of HS and M-MA. ZJ, SL, HZ, H-QD, and W-TH synthesized and characterized the nanoparticles under the mentorship of Y-YL. ZJ, X-RQ, and Y-FS performed the animal experiments and analyzed the results. Y-YL’s team analyzed the material data and wrote the corresponding portion of the manuscript. M-MA’s team (Y-TD, SL and JL) analyzed the cellular data and wrote the corresponding portion of the manuscript. HS’s team analyzed the animals’ data and wrote the corresponding portion of the manuscript. ZJ, Y-TD, SL and JL finished revising the manuscript together. All authors read and approved the final manuscript.

## Funding

This study was supported by he National Key Research and Development Program of China (2021YFC2300400), the Shanghai Natural Science Foundation (20ZR1459500) and the National Natural Science Foundation of China (82173864, 81601745, 81671989).

## Conflict of Interest

The authors declare that the research was conducted in the absence of any commercial or financial relationships that could be construed as a potential conflict of interest.

## Publisher’s Note

All claims expressed in this article are solely those of the authors and do not necessarily represent those of their affiliated organizations, or those of the publisher, the editors and the reviewers. Any product that may be evaluated in this article, or claim that may be made by its manufacturer, is not guaranteed or endorsed by the publisher.
